# VOCs采集与分析技术在肺癌诊疗中的研究进展

**DOI:** 10.3779/j.issn.1009-3419.2021.101.41

**Published:** 2021-11-20

**Authors:** 玲 郭, 红 邬, 强 李, 川 许, 羽阳 刘

**Affiliations:** 1 610041 四川，电子科技大学医学院附属肿瘤医院/四川省肿瘤医院 Department of Sichuan Cancer Hospital & Institute, Sichuan Cancer Center, School of Medicine, University of Electronic Science and Technology of China, Chengdu 610041, China; 2 100853 北京，解放军医学院 Medical School of Chinese PLA, Beijing 100853, China

**Keywords:** 肺肿瘤, 挥发性有机化合物, 采集与分析, 早期诊断, Lung neoplasms, Volatile organic compounds, Collection and analysis, Early diagnosis

## Abstract

肺癌是全球范围内发病率和死亡率最高，对人群健康和生命威胁最大的恶性肿瘤之一。筛查与早诊早治是降低人群肺癌死亡率的有效措施。挥发性有机化合物（volatile organic compounds, VOCs）检测以快捷性、无创性、采样方便成为一种具有广泛前景的检测手段，可用于肺癌的早期发现，病程检测及预后管理。目前国内外涌现出了多种VOCs采集设备及分析技术，本综述主要从VOCs采集技术、分析技术以及在肺癌诊疗中的应用研究进展三方面探讨VOCs研究目前所存在的问题及研究前景。

## Abstract (en)

Lung cancer is a leading cause of cancer-related morbidity and mortality globally, which is the biggest menace to the health and life of the population. Screening and early detection of lung cancer are effective in reducing its mortality, and the measurement of volatile organic compounds (VOCs) has become a promising clinical means for early detection, course detection and prognosis management of lung cancer, with advantages of rapid speed, non-invasiveness and convenience. Now, a variety of VOCs collection ways and analysis methods have emerged at home and abroad. This report summarized three aspects, including VOCs collection, multiple methods of analysis and progress in the diagnosis and treatment of lung cancer. At last, we discussed the limitations and prospects of VOCs analysis.

肺癌是全世界癌症致死的主要原因，占癌症死亡总数的18%，在中国，它是男性癌症死亡的主要原因，也是女性癌症死亡的第二大原因。GLOBOCAN2020数据^[1]^分析显示，中国肺癌发病数和死亡数分别占全球的37.0%和39.8%。肺癌患者的5年生存率随着诊断分期的增高而降低，由此可见高危患者早期筛查的重要性^[2]^。目前在各类肺癌筛查指南或共识中，低剂量螺旋计算机断层扫描（low-dose computed tomography, LDCT）为临床上筛查肺癌的主要方式。在我国，LDCT目前用于50岁-74岁的人群的高危筛查。研究^[3]^发现，与未筛查人群相比，LDCT筛查的I期肺癌检出率提高了4.7倍（OR=5.73, 95%CI: 3.37-9.76），而肺癌相关的死亡率降低了24.0%（OR=0.76, 95%CI: 0.66-0.88）。LDCT存在的主要问题有两个方面。一方面是其假阳性率高，美国国家肺癌筛查实验组公布的数据^[4]^显示，使用LDCT进行肺癌筛查的假阳性率高达96.4%，其直接后果就是过度诊断以及随后的过度治疗，即意味着，如果不进行LDCT筛查，一些患者终生都不会被诊断为肺癌；另一方面是检查过程辐射的危害，研究^[5]^发现在10年的LDCT筛查中诊断出的每108例肺癌中就有1例患者是在筛查过程中由于辐射引起的。在上述因素的考量下，寻找一种更安全、更便捷的肺癌早期诊断方式成了当务之急。

中医讲究“望闻问切”，其中闻便是指医生听患者说话、咳嗽、呼吸，用鼻子嗅出患者口腔和各种分泌物的气味。研究^[6]^发现临床中苯丙酮尿症患者尿液，汗液中会散发特殊的鼠尿味，有机磷农药中毒患者呼出气和尿液中会伴有明显的大蒜气味，经过训练的狗可以闻出恶性黑色素瘤患者，这说明机体疾病状态下会产生特殊的挥发性气体。挥发性有机化合物（volatile organic compounds, VOCs）是起源于人体代谢过程的化合物，主要包括烷烃类、烯醇类、醛酮类等。作为一种新型的非侵入性肿瘤生物标志物，被认为可用于包括肺癌、乳腺癌、胃癌、胰腺癌以及结直肠癌等在内的癌症的早期筛查而进入研究者视野^[7-12]^。VOCs在室温下稳定存在，沸点在50 ℃-260 ℃^[13]^，人体细胞通过代谢产生VOCs，实验^[14]^证实，肺癌患者与非肺癌个体的内源性VOCs种类和含量存在差异。体内组织发生癌变时，伴随着癌细胞代谢重编程，线粒体会产生大量的氧自由基，这些氧自由基透过线粒体膜进入胞浆后，可导致蛋白质、脂肪酸以及核苷酸的氧化损伤，例如不饱和脂肪酸的过氧化反应会生成烷烃类物质而在各类体液中被检测到。因此VOCs对肺癌早期诊断具有重大意义。

尽管在肺癌早期诊断中VOCs分析是一种极具发展前景的技术手段，但样品采集、富集等处理方式多样，分析手段繁杂。下面即通过总结在肺癌筛查中患者体液（呼出气、尿液、血液、胸膜腔积液）来源及肿瘤细胞系来源的VOCs采集分析的多种技术方式，就该方法当前的局限性和应用前景进行综合分析和讨论（图 1）。

**图 1:**
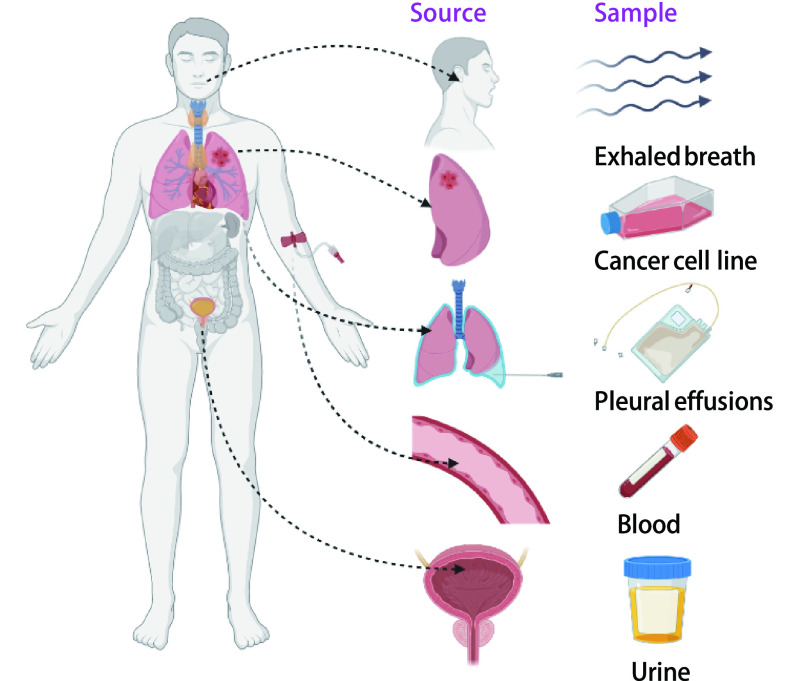
VOCs来源 Sources of VOCs. VOCs: volatile organic compounds.

## 1 VOCs的采集

### 1.1 患者体液来源

#### 1.1.1 患者体液来源气体状VOCs的采集

人体中VOCs一经形成便可以进入血液，在血液循环中到达肺部，通过肺泡进行气体交换从而排出体外^[13]^。近年来呼出时的若干种挥发性碳基化合物已被鉴定为癌症特异性标记物，收集及分析其中代表机体（包括肿瘤细胞、微生物）代谢状态的内源性气体化合物的组成，对于肺癌患者早期诊断具有重要意义。呼出气采集是无创的，采样量、采样时间、采样频率不受限制，具有良好的可重复性。目前使用的收集方法主要有气袋收集法、Bio-VOC取样器以及采气仪耦合吸附管取样器等。

由于使用便捷、经济，气袋收集法是目前临床上使用最多的一种方法。受试者需佩戴鼻夹，打开进气阀门后通过一次嘴呼吸将1 L左右呼出气采集入取样袋，封闭取样袋送往实验室进行分离检测。目前使用最多的是Tedlar气袋采样，该气袋的聚氟乙烯材质比其他化合物具有更大的化学结合力与稳定性。研究^[15]^表明，与Kynar及Flexfifilm等其他类型取样袋相比，Tedlar袋具有背景污染低、样品稳定性高（干燥样品可保存7 d）和可重复性的优势。Bio-VOC取样器主要用于采集肺泡内气体，可以排除呼出气中与机体代谢无关的死腔气体的影响，受试者缓慢呼出约450 mL气体进入聚氟乙烯取样器，前半段呼气被排出，后半段约150 mL气体被保留用于后续检测^[16]^。该方法操作简单，易于携带及保存，但存在样品采集量低、分析难度大的问题。同样的，采气仪耦合吸附管取样器主要是利用采气仪采集呼出气后直接进入到装填有Tenax的吸附管内，可以实现其中有机化合物的直接吸附，同时也可以排除死腔气体的影响，但操作繁琐，成本相对较高。

相对应的，不同的采集方式收集的气体有不同的富集方式，气袋收集法与Bio-VOC取样器收集的气体采用固相微萃取，其中气袋收集法也可直接进样分析。采气仪富集的VOCs后续经热脱附仪（thermal desorption, TD）进行浓缩提纯，其中热脱附仪是对吸附在Tenax管中的物质通过高温加热进行脱附处理，然后吹扫进后续的分析系统中^[17]^。

#### 1.1.2 患者体液来源液体状VOCs的采集

呼出气冷凝物（exhaled breath condensate, EBC）是指将呼出气进行液化收集而来的液体，也是一种很有研究前景的肺部疾病标记物来源。与传统收集呼吸道液体（痰、支气管肺泡灌洗液等）方法相比，具有无创、实时、简便、可重复、不会改变呼吸道的环境等特点。目前临床上绝大多数EBC收集装置为商业化收集器，包括R-Tube、EcoScreen、TURBO-DECCS和ANACON。使用冷凝器采集受试者呼吸15 min的样品，在采集程序结束之前每5 min检查一次呼吸频率和平均呼吸量。收集到的EBC样品立即在冰上运到实验室。然后将样品转移至2 mL管，储存在-70 ℃用于后续分析。德国Eric Jaeger公司生产的EcoScreen冷凝器能够维持样品收集过程中-20 ℃恒低温，有明显的应用优势^[18]^。

患者体内尿液、血液、胸腔积液等也是VOCs的重要来源，与呼出气相比，这类体液中VOCs的采集方式相对简单，但往往需要富集的过程。目前采用的富集装置主要是微萃取（microextraction），微萃取按萃取相分为液相微萃取（liquid phase microextraction, LPME）和固相微萃取（solid-phase microextraction, SPME）。1996年以来，LPME随着环境分析技术的迅速发展应运而生，其基本原理是以有机液滴为萃取剂，在样本和萃取剂两者间使目标分析物分配达到平衡，从而萃取出目标溶质。LPME具有快速、准确、灵敏度高、环境友好的显著优势。SPME技术是由Pawliszyn在20世纪90年代初提出，由液固萃取以及液相色谱相结合发展而来的新型样品富集技术。SPME以吸附剂为核心，液体样品通过吸附剂后，保留下了被测物质。在去除杂质后即用少量溶剂洗脱被测物质以达到快速分离、纯化和浓缩的目的。SPME中常用的固体吸附剂有Tendax管、活性炭管及混合吸附剂等。此外，研究者利用新型金属有机框架材料MOF-5作为吸附剂进行现场取样富集甲醛，并与TD-气相色谱/质谱法（gas chromatography/mass spectrometry, GC/MS）联用具有线性范围宽、灵敏度高、再现性好、不需要化学衍生等优点^[19]^。

尿液VOCs的富集主要采用Tenax管顶空固相萃取，Ramos等^[20]^采用如下方法，收集受试者尿液储存于-20 ℃，常温融化，然后移入15 mL带螺帽玻璃离心管中5, 000 rpm离心10 min。之后将每种尿液4.0 mL与2.0 g氯化钠以及50 mL超纯水转移入10 mL的Teflon^®^/硅胶隔密封样本瓶。血液样本经收集后，一般使用HS-SDME进行VOCs富集，即有机溶剂液滴悬浮在进样针或毛细管端口上，在样品上方悬空进行萃取。例如使用衍化剂PFBHA的悬浮微滴溶剂提取、浓缩并衍生血液中的醛，这种萃取方式可以避免收到样本基质中高分子化合物以及难挥发物质的污染。也有研究将超声联合HS-LDME萃取，大大提高了有机溶剂的稳定性和方法的灵敏度，此类方法具有简便、灵敏、高效、溶剂消耗低、样品基质干扰小等优点。它为研究复杂生物样品中的挥发性疾病生物标志物（醛类）提供了巨大的潜力^[21]^。胸腔积液作为与肺组织直接接触的样本，可能比血液、尿液样本肺癌相关VOCs含量更高。采用胸腔穿刺术采集并储存样品在-80 ℃，Liu等^[22]^使用固相微萃取HS-SPME联合气相色谱质谱法GC-MS研究肺癌患者胸腔积液中VOCs，研究结果表明，HS-SPME-GC/MS是一种简单、快速、敏感、无需溶剂的胸腔积液样本测定方法，胸腔积液是区别肺癌患者和炎症个体VOCs的有价值的样本来源。

### 1.2 肿瘤细胞系来源气体状VOCs的收集

人体VOCs的产生不仅仅依赖于肿瘤细胞，其他正常细胞、免疫细胞和感染性病原体也会产生挥发性有机化合物，而且在采集分析过程中容易受到患者年龄、性别、饮食、吸烟等影响很难统一标准化。因此，分析来自肿瘤细胞系的顶空化合物可以识别肿瘤特异性的挥发性有机物，并有助于阐释其生物起源，而且作为体外研究，比体内研究更容易控制实验变量，排除如性别、年龄和个体间变异的影响（原代细胞除外）。实验中一般用细胞培养瓶培养细胞，检查前24 h更换培养液，并隔绝与外界气体交换，为排除细胞培养液挥发气体的影响，将空白培养基设置为阴性对照。随后采用SPME收集培养瓶内混合气体并予以浓缩随后分析。Schmidt等^[23]^采用吹扫捕集法（purge technique, PT）收集体外细胞培养VOCs，该方法又称为动态顶空萃取法。两种方法集吸附、富集、解吸、进样于一体，通常与GC/MS等仪器联用。

在进入分析仪之前的进样方式上，目前研究中使用较多的为程序升温气化进样（programmable temperature vaporizing, PTV）。顾名思义，程序升温气化进样一般是在低温条件（例如35 ℃）下将样品注射进样口称管内，继而按照设定的程序逐步升高进样口温度（例如250 ℃），使样品迅速气化^[20]^，PTV可以把分流进样、不分流进样和冷柱进样结合为一体，适用范围广，灵活性高。PTV通常与GC、MS等联合用于VOCs的成分分析（表 1）。

**表 1:** 富集方式及特点 Collection methods and characteristics

Microextraction	Classification	Characteristics
TD	-	Suitable for samples which are adsorbed in adsorption tube
LPME	SDME	Direct contact with sample
	HF-LPME	In-direct with sample
	HS-SDME	Suitable for volatile and semi-volatile samples
SPME	Direct SPME	Direct contact with sample
	HS-SPME	Suitable for volatile and semi-volatile samples
	MP-SPME	Protect stationary phase
TD: thermal desorption; SDME: single drop microextraction; HF-LPME: hollow fiber liquid-phase microextraction; HS-SDME: headspace single drop microextraction; Direct SPME: direct solid-phase microextraction; HS-SPME: headspace SPME; MP-SPME: membrane-protected SPME.		

## 2 VOCs的分析

在大多数情况下，生物体来源的VOCs组成复杂，传统分析方式效果不佳。因此，实现生物体挥发性有机物成分的定性和定量分析主要依赖于高通量分析技术。生物挥发性有机物分析技术主要分为包括色谱法（chromatography）、质谱法（mass spectrometry, MS）以及电子鼻（electronic nose, E-nose）和传感器技术相互间的组合与衍生。

### 2.1 色谱法

色谱法又称色层法，主要由固体相以及流动相两部分组成。按流动相分为液相色谱法、气相色谱法等。基本原理是以液/气体为流动相，将不同极性的单一溶剂或不同比例的混合溶剂、缓冲液或汽化的试样被载气等流动相泵入装有固定相的色谱柱，各成分在色谱柱内被分离后进入检测器，采用适当的鉴别和记录系统生成色谱图，标明各组份流出色谱柱的时间和浓度，从而实现对样本的分析。目前应用较多的色谱法主要为高效液相色谱法（high performance liquid chromatography, HPLC），因其采用高压输液系统故又称高压液相色谱。此外超高效液相色谱法（ultra performance liquid chromatography, UPLC）采用小颗粒，高性能微粒固定相，改善了色谱的分离度、灵敏度和样品通量。多维气相色谱的发展大大提高了复杂生物挥发性有机物的分离能力，相比单维气相色谱更容易得到VOCs潜在的生物信息。色谱作为一种分离技术必须联合其他的检测系统才能对各组分进行定量检测，其偶联的检测系统主要包括氢火焰离子化检测器（flame ionization detector, FID）以及MS^[24]^。相比GC-FID，GC-MS优势在于结构注释功能佳、灵敏度高和速度快，是检测呼出气中挥发性有机化合物最常用的仪器^[25, 26]^。

### 2.2 MS

MS是依据离子质荷比（质量-电荷比，m/z）来检测样品的技术，即运动的离子通过电场和磁场根据不同的质荷比分离开来，通过测出离子准确质量即可确定离子的化合物组成。

#### 2.2.1 选择性离子流管质谱（selected ion flow tube MS, SIFT-MS）

SIFT-MS检测空气或者呼出气中痕量化合物的实时量化分析技术，基本原理是流动管技术，化学离子反应和直接质谱法，将初始离子（比如H_3_O^+^、NO^+^、O_2_^+^等）和由载气导入的痕量气体在一定时间内进行化学电离。SIFT-MS通过快速实时在线分析，可快速定量分析浓度低至pptv级别的VOCs和一些无机气体，且无须对样品进行吸附解吸^[27]^。

#### 2.2.2 质子转移反应质谱（proton transfer reaction MS, PTR-MS）

PTR-MS是基于离子流动管质谱，结合化学电离和流动漂移管模型技术的新型VOCs在线检测技术^[17]^，其本质是一种化学电离质谱法。空心阴极对水蒸气进行放电，产生高纯度的H_3_O^+^离子进入反应室，在其中引入要分析的空气样品。初始离子H_3_O^+^通过质子转移反应将质子转移给待测分子使其离子化，随即利用质谱检测技术测定有机物的绝对浓度。PTR-MS优势在于可以在线实时监测上千种挥发性有机物，无需样品预处理，不仅可以通过直接进样检测气态样品，结合顶空进样可分析液态或固态形式中所含的挥发性有机物^[28]^。

#### 2.2.3 飞行时间质谱（time of flight MS, TOF-MS）

离子在一定距离真空无场区内到达检测器的时间会因为离子质荷差异而不同，TOF-MS技术正是通过这个不同时间而建立质谱图。经典TOF-MS由三个主要结构即离子源、飞行管、检测器及两个系统即记录系统和真空系统组成。相较传统质谱仪，它的优势在于结构简单、离子流通率高且质量范围不受限制。Rudnicka等^[29]^将TOF-MS与GS联合用于生物样品的预浓缩、分离和分析，可以用于快速测定呼出气中ppb级别的化合物。

#### 2.2.4 傅立叶变换离子回旋共振质谱（fourier transform ion cyclotron resonance, FT-ICR）

FT-ICR-MS将傅立叶变换应用于离子回旋共振质谱分析，离子源产生离子束并引入ICR中，离子在宽频域射频信号（包涵所有离子回旋频率）作用下进行回旋运动，类似电流的信号在记录在接收板上。FT-ICR-MS集超高分辨率，高灵敏度和超高质量精度（< 200 ppb）等优良性能为一体。研究^[30]^证明使用FT-ICR-MS进行VOCs分析在区分肺癌患者与吸烟者以及非吸烟者均有较高的敏感度与特异度，在区分肺癌患者以及肺良性结节患者亦有一定预测价值。

### 2.3 电子鼻和传感器技术

电子鼻技术是目前检测呼出气最有发展前景的检测方法，它是一种通过模拟动物嗅觉器官开发出的精密人工嗅觉仪器，主要使用气味指纹图谱对VOCs进行定性或定量分析。自1982年，英国沃里克大学的Persaud等^[31]^提出了具有气体识别、检测、分析功能的仿生“电子鼻”，到近年来Chen等^[32]^利用金属离子诱导氧化石墨烯组装电子鼻诊断肺癌，可以达到95.8%的灵敏度。Chen等^[33]^使用自主研发的电子鼻系统对肺癌的检测和分期进行了研究，对肺癌的识别准确率可达93.59%，肺癌分期的识别准确率达到80%以上。这些研究结果提示了电子鼻检测肺癌VOCs的可行性。电子鼻主要由气味取样器、传感器和信号处理系统组成，分别模拟的是哺乳动物嗅觉系统内嗅上皮、嗅球、嗅皮层三级神经元。当某种VOCs混合物与特定传感器接触并发生反应，输入的化学信号转换成电信号，由多个传感器对一种VOCs混合物的整体响应便构成了传感器阵列对该混合物的整体响应谱，而整个传感器阵列对不同VOCs混合物的响应图谱不同，正是这种区别，使得电子鼻系统可以根据传感器阵列的响应图谱进行不同VOCs混合物的精准识别^[34]^。与传统的GC-MS检测相比，基于传感器检测技术的电子鼻是一种新型检测设备，虽然检测物的范围受限于传感器类型，但是检测效率方面有较大提升（表 2）。

**表 2:** VOCs分析方法 VOCs analysis methods

Sources	Analysis methods
Breathe	GC-MS, SIFT-MS, PTR-MS, FT-ICR-MS, E-nose
Urine	GC-MS, PTV-MS, HS-PTV-MS, PTV-GC-MS, HS-PTV-GC-MS, HS-SPME, GC-TOF -MS
Blood	GC-MS, HPLC, HS-SDME-GC-MS
Pleural effusions	HS-SPME-GC-MS
Lung cancer cell line	GC-MS, SPME-GC-MS, PT-GC-MS, TD-GC-MS, SIFT-MS, PTR-MS, E-nose
GC: gas chromatography; MS: mass spectrometry; FT-ICR: fourier transform ion cyclotron resonance; E-nose: electronic nose; SIFT: selected ion flow tube; PTV: programmable temperature vaporizing; PTR: proton transfer reaction; TOF: time of flight; HPLC: high performance liquid chromatography; PT: purge technique.	

## 3 讨论与展望

肺癌的早期诊断可以显著提高患者的5年生存率。传统的检查方法，如血清肿瘤标志物、痰细胞学以及X线检查，假阳性率较高且检查结果多为中晚期，无法做到肿瘤的早期筛查，CT扫描不能精准分析肿块的性质，造成大量假阳性结果和不必要的组织活检^[35-37]^。人体挥发性有机化合物VOCs可实时反映人体代谢状态，对机体各种来源VOCs（尤其是呼出气VOCs）进行分析，在肺癌早期诊断和治疗监测乃至预后评估中具有重要价值。CT联合VOCs对肺癌进行早期非侵入性诊断，可以达到100%的特异度与97.3%的灵敏度^[38]^。VOCs因其无创、操作简便等特点，将使其成为患者及医生的首要选择。

现有的VOCs采集、富集、成分分析、统计评估技术都相对成熟，但也不可避免的存在一些问题。①胸腔积液、血液采集过程的相对有创，因此我们更多的选用呼出气VOCs进行肺癌早期诊断。但不可否认的是即使相对有创，胸腔积液、血液VOCs的分析也会是低剂量螺旋CT检查的有利补充；②VOCs采集与分析过程的标准化，不同研究者之间因为实验客观条件的制约，尚未形成统一检测标准从而导致难以进行横向比较。采用传感器为基础的检测设备来检测具体的标志物是一种选择趋势，不仅速度快，而且费用低，同时也能保证一定的准确率；③已提出的可能作为肺癌的生物标记物起源不明，不同体液之间差别较大。比较肺癌的不同个体、同一个体的不同体液，找到其中的尤其是体外肺癌细胞系与体内肺癌细胞代谢产生的VOCs之间的共同点，并尝试从肺癌起源代谢通路找到证实依据，排除人体非肿瘤源性挥发性有机化合物的影响，从而发现肺癌的特异性生物标记物。

VOCs作为近年新兴的肺癌检测方式，发展潜力巨大。VOCs研究起步相较传统方法晚，发展时间有限，除在胰腺癌检测中进行了前瞻性实验研究^[9]^，近5年在肺癌检测中尚未有前瞻性实验报道，因此该项研究需要多学科多领域通力合作，产学研一体化发展，促进科研成果向临床快速转化。VOCs联合传统肺癌早期检测方式，有望提高早期检测肺癌患者的能力，改善肺结节的管理模式，降低筛查和诊断风险和成本，优化预后监测方法，同时会成为临床上疾病诊断（表 3），包括其他癌症、糖尿病、神经系统疾病等的主要检测方式之一，惠及更多患者。

**表 3:** 患者体液及肺癌细胞系的VOCs研究总结 Studies on the VOCs analysis of patient-derived body fluids and lung cancer cell lines

Sources	Study	Collection	Control groups	Analysis methods	Efficiency				Summary
					Sensi- tivity (%)	Speci- ficity (%)	AUC	Other	
Breathe	2021^[33]^	Tedlar bag	Healthy	E-nose	95.6	91.1	-		New technology and prospective way
	2019^[39]^	Tedlar bag	Healthy	E-nose	96.2	90.6	­-		New technology and prospective way
	2019^[40]^	Tedlar bag	Healthy	GC-MS	80	91.23	­-		Typical
	2019^[41]^	Tedlar bag	Healthy	GC-MS	75.4	85	­-		Typical
	2017^[42]^	Tedlar bag	Healthy	GC-MS	95	89	-		Typical
	2015^[43]^	Tedlar bag	Healthy	GC-MS	63.5	72.4	0.65		Typical
	2015^[44]^	Tedlar bag	Healthy benign	FT-ICR-MS GC-MS	96	64^a^ 86^b^ 100^c^	0.96		High sensitivity and mass accuracy
	2014^[45]^	Tedlar bag	Healthy benign	FT-ICR-MS	28	100	0.86		Higher specificity than PET in distinguishing benign disease
	2021^[11]^	Tedlar bag	Healthy	SIFT-MS	96	88	0.98		A persuasive platform for lung cancer prediction
	2016^[46]^	Tedlar bag	Healthy	PTR-MS	-	-	-		Cannot identify compounds with certainty
	2009^[47]^	Tedlar bag	Healthy	PTR-MS GC-MS	80	100	-		Does not need preconcentr-ation, more reliable quant- itative results
Urine	2017^[20]^	SPME	Healthy	HS-PTV-MS	100	100	-		High sensitivity and specificity, insufficient samples
Blood	2010^[48]^	Ultrasound-assisted HS-SDME	Healthy	HPLC		-	-	Some alkanes are high	Simplicity, low cost and short sample preparation time
Pleural effusions	2014^[22]^	HS-SPME	Healthy	GC-MS	-	-	-	*P* < 0.05	Simple, rapid, sensitive and solvent-free
Cancer cell line A549 Calu3	2018^[49]^	SPME	MCF7 WI38VA13	GC-MS E-nose	-	-	-	Some alkanes are high	Non-invasive diagnostic tool to classify lung cancer
^a^: lung cancer *vs* patients with benign nodules; ^b^: lung cancer *vs* smokers; c: lung cancer *vs* non-smokers; AUC: area under the curve; PET: positron emission tomography.									
